# Analysis of simple sequence repeat (SSR) structure and sequence within *Epichloë* endophyte genomes reveals impacts on gene structure and insights into ancestral hybridization events

**DOI:** 10.1371/journal.pone.0183748

**Published:** 2017-09-08

**Authors:** William Clayton, Carla Jane Eaton, Pierre-Yves Dupont, Tim Gillanders, Nick Cameron, Sanjay Saikia, Barry Scott

**Affiliations:** 1 Institute of Fundamental Sciences, Massey University, Palmerston North, New Zealand; 2 The Bio-Protection Research Centre, Massey University, Palmerston North, New Zealand; 3 Cropmark Seeds Ltd, Darfield, New Zealand; Ruhr-Universitat Bochum, GERMANY

## Abstract

*Epichloë* grass endophytes comprise a group of filamentous fungi of both sexual and asexual species. Known for the beneficial characteristics they endow upon their grass hosts, the identification of these endophyte species has been of great interest agronomically and scientifically. The use of simple sequence repeat loci and the variation in repeat elements has been used to rapidly identify endophyte species and strains, however, little is known of how the structure of repeat elements changes between species and strains, and where these repeat elements are located in the fungal genome. We report on an in-depth analysis of the structure and genomic location of the simple sequence repeat locus B10, commonly used for *Epichloë* endophyte species identification. The B10 repeat was found to be located within an exon of a putative bZIP transcription factor, suggesting possible impacts on polypeptide sequence and thus protein function. Analysis of this repeat in the asexual endophyte hybrid *Epichloë uncinata* revealed that the structure of B10 alleles reflects the ancestral species that hybridized to give rise to this species. Understanding the structure and sequence of these simple sequence repeats provides a useful set of tools for readily distinguishing strains and for gaining insights into the ancestral species that have undergone hybridization events.

## Introduction

Most agriculturally important cool season grasses contain endophytic fungi of the genus *Epichloë* (Ascomycota, Clavipitaceae) [1,2]. These fungi systemically colonize the intercelluar spaces of leaves of both vegetative and reproductive tissues and confer on the host protection from various biotic and abiotic stresses, thereby leading to greater persistence in the field [2,3]. The best documented of these benefits is increased resistance to insect herbivory due to the production of secondary metabolites such as peramine and lolines that reduce damage to the pastures [4,5,6]. However, use of endophytes in pastoral systems can also result in the production of anti-mammalian metabolites, which cause problems in grazing livestock such as fescue toxicosis and ryegrass staggers [7,8].

Endophyte species were previously defined on the basis of morphology and host specificity [9,10], but more robust methods for identification were subsequently developed using molecular phylogenetic analysis of intron sequences from the β-tubulin (*tubB*), translation elongation factor 1-α (*tefA*) and γ-actin (*actA*) genes which allowed for more distinct taxonomic groupings and identification [11,12]. Using these methods the taxonomy of both the sexual and asexual *Epichloë* species were resolved [11,12]. A key finding from these studies was the demonstration that many of the asexual *Epichloë* species are interspecific hybrids [11,13,14]. However, for rapid endophyte strain identification *in planta* a PCR method based on polymorphic simple sequence repeat (SSR) loci was developed [15]. The utility of this method was further improved by the identification of additional SSR loci and development of a multiplex PCR system for strain identification [16].

SSRs, also known as microsatellites, consist of repetitive DNA where short DNA motifs are repeated in tandem to form different lengths of repetitive sequence [17,18]. SSRs arise through slippage of the DNA polymerase during DNA synthesis or repair, thereby giving rise to an increase or decrease in the repeat number [19]. The high variability in repeat numbers makes these loci ideal for use in genetic studies in a wide range of eukaryotes, including plant and fungal species [17,20,21]. SSRs have also been found within gene regions and may play important roles in genetic variation and gene adaptation [22,23,24]. PCR amplification and analysis of SSR loci has been one of the most informative ways of easily identifying endophyte species [16,25].

Previously identified were a set of eleven *Epichloë* SSR sequences (B1 to B11) that proved very useful in a multiplex PCR method to identify and distinguish different *Epichloë* endophyte strains *in planta* [16]. Of the eleven SSRs analyzed, B10 was found to be the most informative for distinguishing different endophyte strains and species by size alone, and when used in combination with other markers such as B11 provided a very powerful and robust system for endophyte identification. This combination of SSRs is by far the most commonly used method by forage grass companies to identify strains of endophytes in their proprietary seeds. While the use of these SSRs has facilitated the rapid identification of different strains, little is known about how the DNA structure of these repeats varies between species and strains and where in the genome these repeats are located.

Given the recent availability of whole genome sequences to many fungi within the family Clavicipitaceae [26], we set out to determine the genome location, sequence and distribution of B10 and related SSRs among these fungi. A further objective was to analyse whether there was sufficient polymorphism in the sequence of B10 to distinguish different ecotypes of the agriculturally important group *E*. *uncinata* [4], a hybrid endophyte of *Festuca pratensis* (meadow fescue) [13], and test its utility in identifying the sexual ancestors of this species.

## Materials and methods

### Fungal strains and growth conditions

Fungal strains used in this study are listed in S1 Table. Liquid cultures were prepared by inoculating 50 ml of potato dextrose (PD) broth with mycelia obtained from plate culture. Cultures were incubated for 7 to 14 days at 22°C on a rotary shaker at 200 rpm.

### Molecular biology methods

Genomic DNA was isolated from freeze-dried mycelium using the method described previously [27]. PCR amplification of SSRs was carried out with the proofreading Phusion^®^ High-Fidelity DNA polymerase (Thermo Scientific). The primers used in this study are listed in S2 Table. PCR products were cloned into the *Escherichia coli* plasmid vector pGEM^®^-T Easy (Promega) as per manufacturer’s instructions. Plasmids were transformed into *E*.*coli* DH5α chemically competent cells, the plasmids purified and the sequences of the SSRs determined. DNA sequencing was performed by the Massey Genome Service using BigDye^TM^ Terminator (version 3.1) Ready Reaction Cycle Sequencing Kit (Applied Biosystems). Sequence analysis was performed using MacVector® version 10.0.2 (MacVector Inc.).

### RNA isolation and RT-PCR

Total RNA was extracted from *Epichloë typhina* E8 mycelia using TRIzol^®^ reagent (Invitrogen). Approximately 1 g of mycelium was ground in liquid nitrogen in a mortar and pestle before addition of 1 ml of TRIzol^®^. The mixture was allowed to thaw at room temperature before being transferred to a 15 ml tube and centrifuged at 9,700 rpm at 4°C for 10 minutes. The supernatant was transferred to a fresh tube and 200 μl of chloroform was added. The solution was mixed thoroughly and allowed to sit at room temperature for 3 minutes before being centrifuged at 9,700 rpm at 4°C for 15 minutes. The aqueous phase was then transferred to a new tube and 500 μl of isopropanol was added followed by incubation at room temperature for 10 minutes to allow the RNA to precipitate. Samples were then centrifuged at 9,700 rpm at 4°C for 10 minutes. The supernatant was discarded and 1 ml of 75% ethanol was added to the RNA pellet before being centrifuged at 6,700 rpm at 4°C for 5 minutes. The RNA pellet was air-dried and re-suspended in 100 μl of diethyl pyrocarbonate-treated water for further analysis.

One μg of *E*. *typhina* total RNA was heat denatured and reverse transcribed using SuperScript^TM^ II RT (Invitrogen) according to the manufacturer’s instructions. cDNA was then used as a template for PCR specific to genes of interest.

## Sequence analysis

SSR sequences from the Clavicipitaceae family were obtained from the University of Kentucky Genome Projects site (http://www.endophyte.uky.edu/). Sequence of *Epichloë bromicola* E799 was obtained through direct sequencing. Sequence comparison was performed using ClustalW within MacVector^TM^ using default settings. Sequences (CAG)_5_(CAT)_5_, (CAG)_5_(CAT)_5_(CAA)_5_ and (CAG)_5_(CAA)_5_ were used as queries in BLASTn search of the *E*. *typhina* E8 and *Epichloë festucae* Fl1 genomes in order to identify B10-like repeat regions.

### Phylogeny reconstruction

To reconstruct the phylogeny of *Epichloë* spp. B10 alleles, the B10 sequences of *Epichloë* spp. were obtained from the University of Kentucky Genome Projects site (http://www.endophyte.uky.edu/) as well as through direct sequencing (for *E*. *uncinata* U2, U3, U4, U5, U6, U7, U9, U10, U12 and U13 strains). The sequences were aligned using MAFFT v7.273 software [28] with the set of parameters linsi [29]. The alignment was then manually verified. The maximum-likelihood phylogeny was reconstructed using PhyML v.2016115 [30] from the ete3toolkit v.3.0.0b35 [31]. The substitution model was chosen using pmodeltest v1.4 from ete3 [31]. Approximate likelihood ratio tests were computed as branch supports. The tree was edited on the interactive Tree Of Life (iTOL) web site [32,33].

## Results

### *Epichloë* B10 SSR lies within an exon of a putative bZIP transcription factor

**Fig 1:**
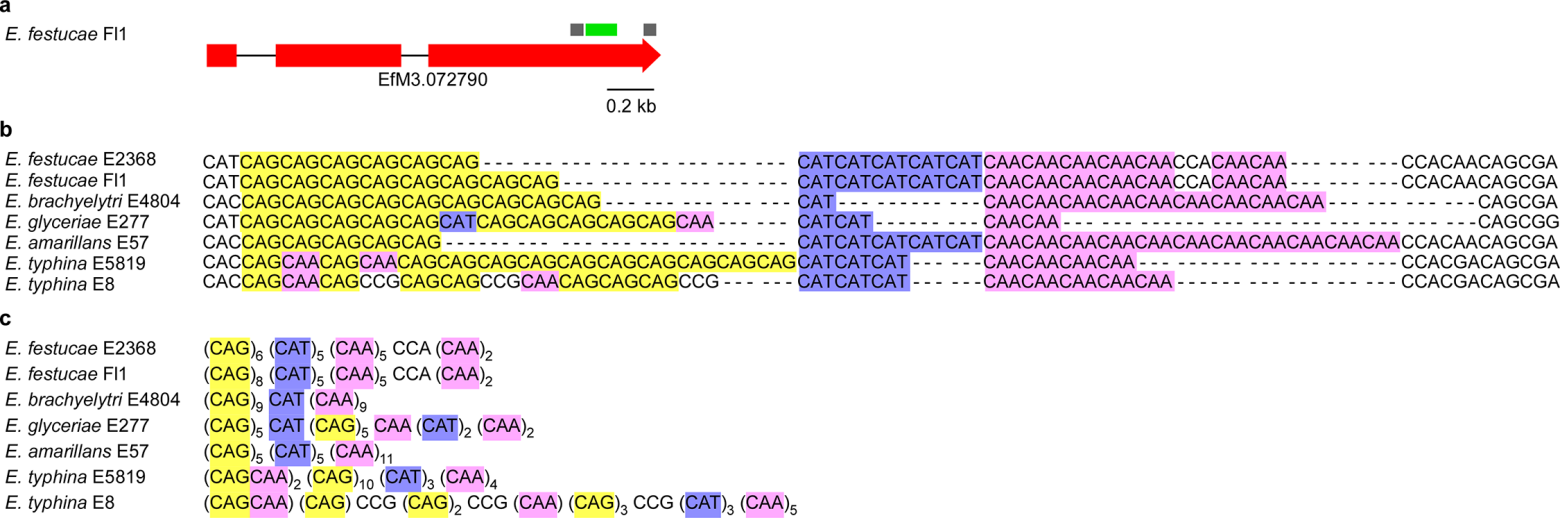
Overview of B10 SSR. (a) Genomic region containing the B10 SSR in *Epichloë festucae* Fl1. The position of primers B10.1 and B10.2 used to amplify the B10 SSR (green) are shown in grey. The SSR is within an exon of a putative bZIP transcription factor (EfM3.072790) (red). (b) Repeat sequence of (CAG)_n_(CAT)_n_(CAA)_n_ between *Epichloë* strains is colored by tri-nucleotide repeats, CAG (yellow), CAT (violet), CAA (pink). Single nucleotide polymorphisms resulting in changes in tri-nucleotide repeats (CCG and CCA) are not colored. (c) Consensus sequences for each *Epichloë* strain identifying variety in repeat units.

**Fig 2:**
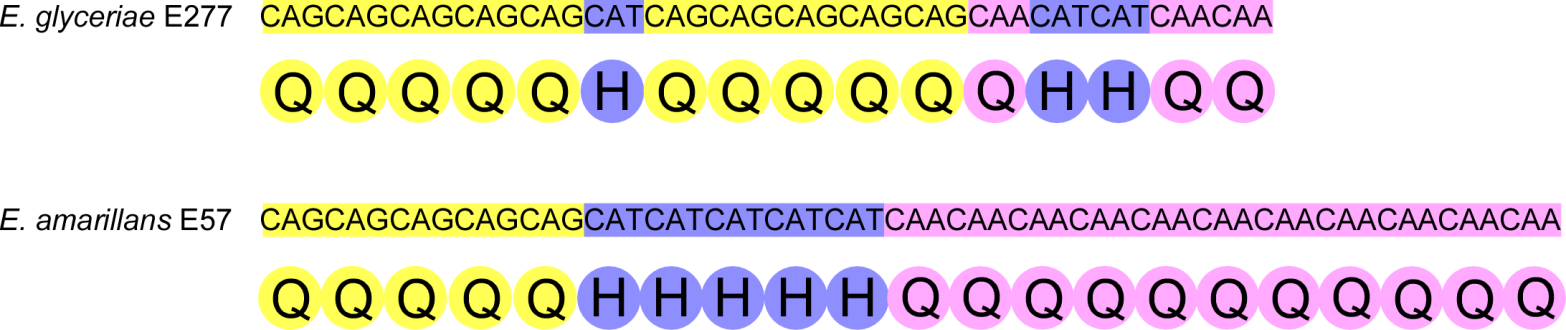
Consequences of B10 repeat changes on peptide sequence. Comparison of the B10 repeats of *Epichloë glyceriae* and *Epichloë amarillans* and the amino acid each tri-nucleotide encodes. Repeats are colored CAG (yellow), CAT (violet) and CAA (pink). Amino acids encoded are colored for the repeat sequence from which they are derived.

Given B10 SSR has been commonly used as a polymorphic marker to distinguish different *Epichloë* endophyte strains [16], we analyzed its sequences from different *Epichloë* genomes for localization within the genome and the repeat structure. This analysis showed that the B10 SSR was present within the third exon of a putative bZIP transcription factor (Gene model EfM3.072790) [26]) and was highly polymorphic (Fig 1A). The B10 repeat comprised of three distinct tri-nucleotide repeats, CAG, CAT and CAA, which conferred a unique polymorphic structure for each species analyzed (Fig 1B). The two *E*. *festucae* strains, Fl1 and E2368, analyzed had repeat sequences that were identical, except for an expansion of two tri-nucleotide CAG repeats found within strain Fl1, which were absent from strain E2368. While the CAT and CAA core repeat structures were close to homogeneous across the seven strains analyzed, the presence of several single nucleotide polymorphisms (SNPs) within the CAG core region of *E*. *typhina* and *Epichloë glyceriae* gave rise to an additional layer of sequence heterogeneity (Fig 1B and 1C). Virtual translation of the B10 SSR within the exon identified a glutamine repeat with a histidine core. An example of how the SSR can lead to changes in polypeptide sequence was illustrated by a comparison of the repeats in *E*. *glyceriae* and *Epichloë amarillans* (Fig 2).

**Fig 3:**
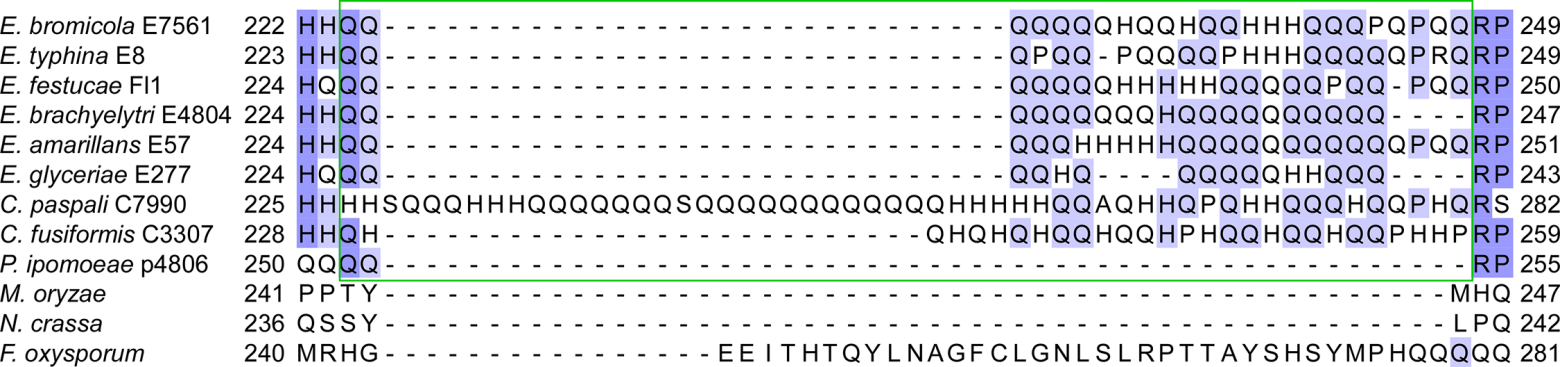
Amino acid sequence alignment of putative bZIP transcription factors. CLUSTALW alignment of amino acid sequences showing the B10 SSR region (green box). The SSR is seen as repeated glutamine (Q) and histidine (H) residues. Gene IDs with associated GenBank protein accession numbers or gene models of protein homologues are: *Epichloë bromicola* EfP3.072790; *Epichloë typhina* EfP3.072790; *Epichloë festucae* EfM3.072790; *Epichloë brachyelytri* EfP3.072790; *Epichloë amarillans* EfP3.072790; *Epichloë glyceriae* EfP3.072790; *Claviceps paspali* EfP3.072790; *Claviceps fusiformis* EfP3.072790; *Periglandula ipomoeae* EfP3.072790; *Magnaporthe oryzae* MGG_08118.6 (XP_003715075); *Neurospora crassa* NCU03847.7 (XP_955781); *Fusarium oxysporum* FOXB_15670.1 (EGU73820).

Analysis of the inferred protein sequence of the putative bZIP transcription factor from *E*. *festucae* Fl1 using SMART (http://smart.embl-heidelberg.de/), predicted the presence of a coiled coil domain between amino acids 39 and 100 that was conserved among other *Epichloë* species and related filamentous fungi (S1 Fig). However, the B10 SSR appeared to be restricted to fungi within the Clavicipitaceae family, including the different *Epichloё* species, the closely related rye pathogen *Claviceps paspali*, and the morning glory endophyte *Periglandula ipomoeae*, but not *Magnaporthe oryzae*, *Neurospora crassa* or *Fusarium oxysporum* (Fig 3). The B10 repeat region in *C*. *paspali* was much larger than that in *E*. *festucae*, whereas the repeat region in *P*. *ipomoeae* was much smaller, and contained few repeated residues (Fig 3). RT-PCR analysis showed that the gene encoding the putative bZIP transcription factor was expressed in *E*. *typhina* strain E8 and cDNA sequencing confirmed the presence of the SSR within the coding sequence (S2 Fig). A size difference of approximately 300 bp between the PCR-amplified gDNA and cDNA sequences confirmed that the two predicted introns were spliced.

### Sequence of B10 alleles in *E*. *uncinata* consistent with an interspecific hybrid origin

**Fig 4:**
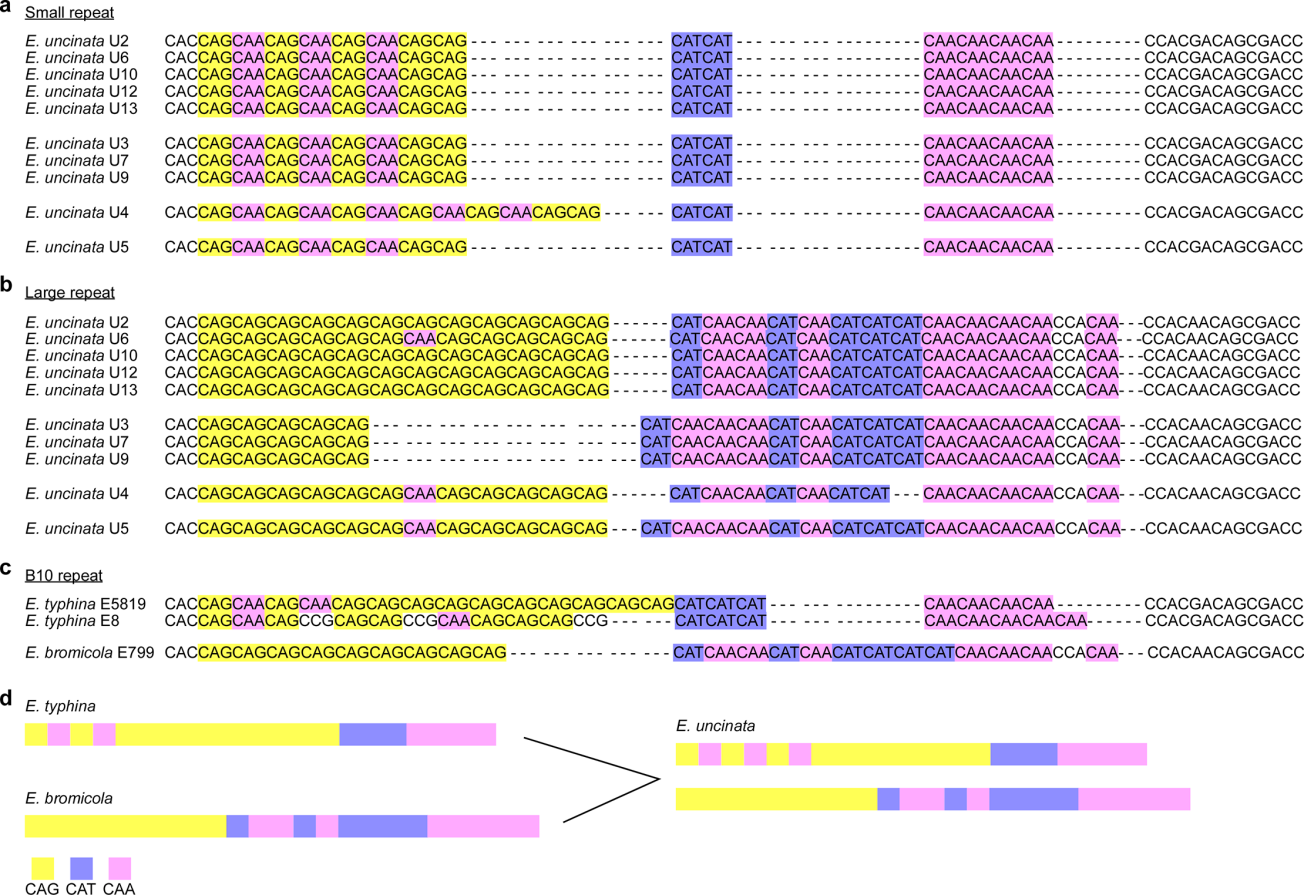
The B10 SSR from *Epichloë uncinata* strains. (a) Sequences of the small B10 repeats contained within each *E*. *uncinata* strain. Strains are grouped and aligned for the different ecotypes. (b) Sequences of the large B10 repeats within *E*. *uncinata* strains. (c) B10 repeats of the proposed ancestors of *E*. *uncinata*, *Epichloë typhina* and *Epichloë bromicola*. (d) Schematic representation of the B10 repeat structures from *E*. *uncinata* and its proposed ancestors. A single B10 allele is found within *E*. *typhina* and *E*. *bromicola*, while two alleles are contained within *E*. *uncinata*. Repeat structure of *E*. *typhina* and *E*. *bromicola* are similar to that of *E*. *uncinata*. Repeat structure is colored to represent repeated units CAG (yellow), CAT (violet) and CAA (pink).

Phylogenetic analysis of *actA*, *tubB* and *tefA* sequences from *E*. *uncinata* indicated that this species is an interspecific hybrid derived from *E*. *typhina* and *E*. *bromicola* [11,13]. Consistent with this hybrid origin was the presence of two copies of the B10 SSR in *E*. *uncinata*. SSR length polymorphism analysis of ten different strains of *E*. *uncinata* separated them into four ecotypes [4], corresponding to the geographic origins of Norway (e.g. ecotype 1 represented by strain U2), Bulgaria (e.g. ecotype 2 represented by strain U3) and Germany (e.g. ecotype 3 and ecotype 4 represented by strains U4 and U5, respectively) (Table 1). To determine whether these strains could be further resolved from one another, the B10 alleles of each of these strains were amplified by PCR, and the two different sized fragments cloned into pGEM^®^-T Easy and sequenced. Sequence analysis of the two B10 alleles from each strain showed that each ecotype had two distinct alleles (Fig 4A and 4B). A close analysis of the B10 allele sequences from these strains revealed that the B10 repeat sequences were polymorphic between the different ecotypes, but conserved within the ecotypes, with the exception of the U6 large allele, which contained a SNP (CAA to CAG) that distinguished it from the other strains in this group (Fig 4B). The repeated units of CAG, CAT and CAA were conserved across ecotypes for the small allele, except in ecotype 3, and were polymorphic across ecotypes for the large allele. There was considerable variation in repeat structure between the large and small alleles. The small allele contained a CAG repeat interspersed with CAA followed by distinct small CAT and CAA repeats. The large allele had a long CAG repeat followed by a mixed CAT and CAA repeat-structure (Fig 4A and 4B). The sequences flanking each allele were highly conserved across all endophyte strains.

**Table 1:** SSR alleles of *Epichloë uncinata and proposed ancestors*.

Species	Strain^1^	B10^2^
*Epichloë typhina*	E8	178.1
*Epichloë bromicola*	ATCC 200750	189.6
*E*. *uncinata* (ecotype 1)	U2, U6, U10, U12, U13	159, 194
*E*. *uncinata* (ecotype 2)	U3, U7, U9	159, 177
*E*. *uncinata* (ecotype 3)	U4	171, 191
*E*. *uncinata* (ecotype 4)	U5	159, 196

^1^Source of *E*. *uncinata* strains: Cropmark Seeds Ltd, Darfield, New Zealand.

^2^PCR products using primers specific for B10. Sizes are in nucleotide units.

**Fig 5:**
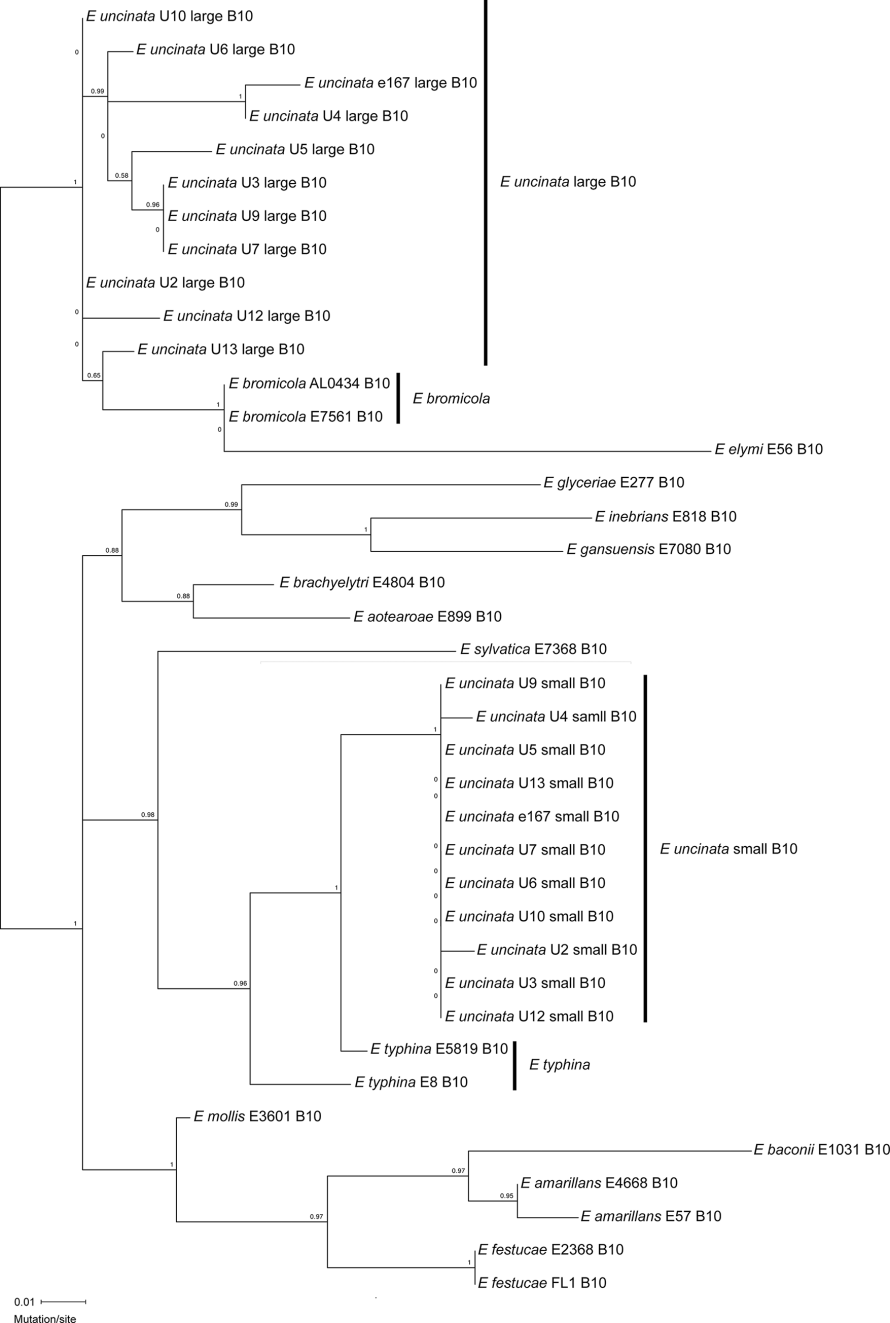
Maximum-likelihood phylogenetic tree of *Epichloë* spp. B10 alleles. The appropriate likelihood ratio test support values are indicated over the branches.

Given *E*. *uncinata* is an interspecific hybrid between *E*. *bromicola* and *E*. *typhina*, the two different B10 alleles observed in *E*. *uncinata* should closely match the single alleles found in each of the parental species. To test this hypothesis, the *E*. *typhina* E5819 and E8 sequences were obtained from available genomic data [26] and the B10 allele of *E*. *bromicola* E799 was sequenced following PCR amplification and cloning into pGEM^®^-T Easy. These sequences were then used for comparison with the *E*. *uncinata* B10 alleles (Fig 4C and 4D). *E*. *typhina* and *E*. *bromicola* each contained a single B10 allele with a structure and sequence very similar to the small length and large length alleles of *E*. *uncinata*, respectively (Fig 4A–4C). The small length B10 allele found in *E*. *uncinata* and the two *E*. *typhina* strains contained CAA repeats within the CAG region. The position of two of the CAA repeats from E5819 matched those of the small length allele in *E*. *uncinata*. The two CCG SNPs found in E8 were not seen in any of the *E*. *uncinata* repeats. The large length B10 allele found in *E*. *uncinata* and *E*. *bromicola* contained distinct regions of mixed CAT and CAA repeats. This region was identical in sequence between ecotype 1 of *E*. *uncinata* and the *E*. *bromicola* allele but the CAG region was different between the two species, with the *E*. *uncinata* ecotype containing an extended CAG repeat of three units. The CAT/CAA region was also very similar in the other ecotypes and matched closely to that of *E*. *bromicola*, however the CAG region did not match any of the strains or ecotypes (Fig 4A–4C). The two different B10 alleles found in *E*. *uncinata* were not found in other available *Epichloë* genome sequences examined (http://www.endophyte.uky.edu/). To further confirm the relatedness between *E*. *uncinata* and its putative ancestors, *E*. *bromicola* and *E*. *typhina*, we carried out a maximum-likelihood analysis of the B10 alleles from various *Epichloë* spp. This analysis showed that the *E*. *uncinata* large and small allele groups were most closely related to *E*. *bromicola* and *E*. *typhina*, respectively (Fig 5).

### Other B10-like repeats in *Epichloë* genomes

**Fig 6:**
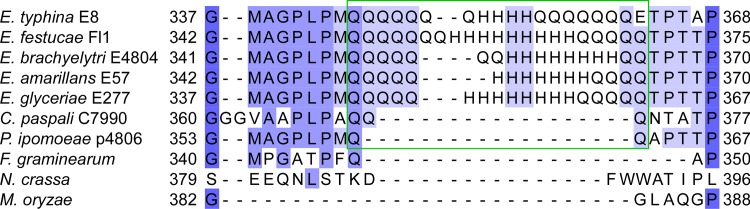
Amino acid sequence alignment of putative copper sensing transcription factors. CLUSTALW alignment of amino acid sequences showing the B12 SSR region (green box). The SSR is seen as repeated glutamine (Q) and histidine (H) residues. Gene IDs with associated GenBank protein accession numbers or gene models of protein homologues are: *Epichloë typhina* EfP3.020790; *Epichloë festucae* EfM3.020790; *Epichloë brachyelytri* EfP3.020790; *Epichloë amarillans* EfP3.020790; *Epichloë glyceriae* EfP3.020790; *Claviceps paspali* EfP3.020790; *Periglandula ipomoeae* EfP3.020790; *Fusarium graminearum* FGSG_08431.3 (XP_011320343); *Neurospora crassa* NCU04773.7 (XP_960222); *Magnaporthe oryzae* MGG_08875.6 (XP_003713900).

During our analysis of the B10 SSR, we found a second B10-like repeat in the *E*. *festucae* Fl1 genome. To follow the convention used previously to describe the SSR repeats B1-B11 [13], we named this SSR as B12. This SSR comprised of a CAG-CAT-CAA repeat and was found within an exon of a gene encoding a putative copper sensing transcription factor (Gene model EfM3.020790) [26]. Analysis of this sequence among other fungal species showed it was polymorphic across the Clavicipitaceae (Fig 6). Protein domain analysis using SMART prediction software revealed a putative copper fist domain (PF00649) at the N-terminus between amino acid residues 4 and 39, while no other functional regions were identified (S3 Fig). With the exception of the repeat region, the amino acid sequence of this protein was highly conserved within the Sordariomycetes (S3 Fig).

To determine the sequence polymorphism of this gene within *E*. *uncinata*, primers were designed to conserved regions adjacent to the SSR and the sequences amplified by PCR. A single product of approximately 300 bp was amplified from each of the different ecotypes. Sequence analysis of this product revealed that there were no differences in this SSR for strains U2, U3, U4 and U5, representative of ecotypes 1 to 4 (S4 Fig). However, sequence analysis of the product from strain U6 (ecotype 1) revealed loss of single CAG and CAA repeats and gain of a single CAT repeat. While the B12 allele in the *E*. *uncinata* strains align best with the *E*. *bromicola* allele there are other possibilities to explain the mutational differences thereby limiting the resolving power of this allele to identify parentage.

**Fig 7:**
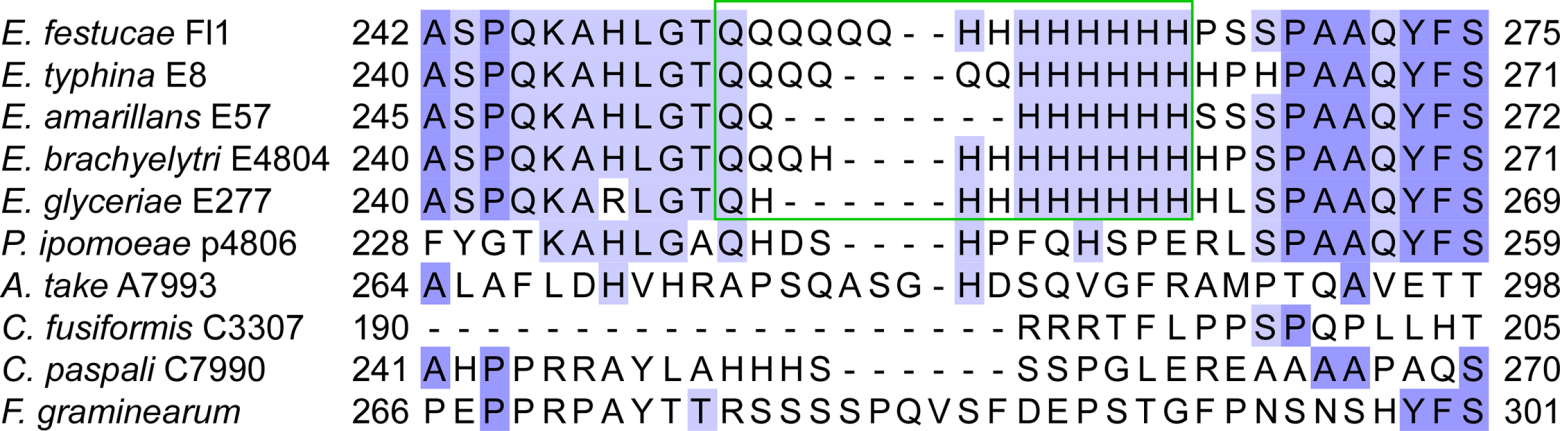
Amino acid sequence alignment of putative G-protein coupled receptors. CLUSTALW alignment of amino acid sequences showing the B13 SSR region (green box). The SSR is seen as repeated glutamine (Q) and histidine (H) residues. Gene IDs with associated GenBank protein accession numbers or gene models of protein homologues are: *Epichloë festucae* EfM3.080640; *Epichloë typhina* EfP3.080640; *Epichloë amarillans* EfP3.080640; *Epichloë brachyelytri* EfP3.080640; *Epichloë glyceriae* EfP3.080640; *Periglandula ipomoeae* EfP3.080640; *Aciculosporium take* EfP3.080640; *Claviceps fusiformis* EfP3.080640; *Claviceps paspali* EfP3.080640; *Fusarium graminearum* FGSG_05239.3 (XP_011323750).

The discovery of an SSR within the exon of a second putative transcription factor prompted us to search the entire *E*. *festucae* Fl1 genome by BLASTn to test if this class of SSR was found within the exons of genes encoding other transcription factors. While additional related SSRs were identified in the exons of other genes there was no specific enrichment in genes encoding transcription factors (Table 2). A typical example was the CAG-CAT repeat found in a putative G-protein coupled receptor, which we named B13 (Fig 7 and S5 Fig). The SSRs identified often coded for a string of glutamine residues but depending on the reading frame and the direction of translation a variety of amino acid repeats were identified. In some genes multiple repeat elements were present that did not consist of repeated glutamine or histidine residues and were derived from sequences other than CAG, CAT or CAA repeats. A BLASTn analysis of other species within the Sordariomycetes, including *M*. *oryzae*, *N*. *crassa*, *Fusarium graminearum*, *C*. *paspali* and *P*. *ipomoeae*, was carried out to determine the distribution of these SSRs among these species. Of the nineteen genes examined, five genes were found to be specific for *Epichloë* (e.g. B13), six for the Clavicipitaceae (*Epichloë*, *Claviceps* and *Periglandula*) whereas the other eight were also identified in *F*. *graminearum*, *M*. *oryzae* and *N*. *crassa* (Table 2).

**Table 2:** B10-like repeats in Sordariomycetes fungi.

Query repeat sequence	Predicted protein containing the repeat	Polypeptide repeats found	Specificity^1^	Identifier
(CAG)_5_(CAT)_5_	G-protein coupled receptor	QQQQQQHHHHHHHPH	*Epichloë*	EfM3.080640
	copper sensing transcription factor	QQQQQQQHHHHQQQQQQQ	*Epichloë*	EfM3.020790
	hypothetical protein	QQQQQQQHHH	*Epichloë*	EfM3.003720
		EDEDEDEDEDEDEDEDEDEDEDE		
	putative GNAT family acetyltransferase	DDDNDDDDNDDNDDDDNDDNDDDDNDDNDDDDDDDD	*Epichloë*	EfM3.068100
	poly A nuclease	QQQQQQQQQQQQHHQQPQQ	Sordariomycetes	EfM3.026960
	cyclin dependent protein kinase complex component	QQQQHQQHQHHHQQHQHHHQQHQQHQHYQQQHH	Sordariomycetes	EfM3.019450
	hypothetical protein	QHQHQHQHQQQQQQHHHHHHQ	Clavicipitaceae	EfM3.045890
(CAG)_5_(CAT)_5_(CAA)_5_	hypothetical protein	YYYYYYYYYYYYYYYYYYYYYYYY	Clavicipitaceae	EfM3.018830
		HNHHSHHHNQQQQQQQQQQQQQQQQQ		
		HHHHHQQQQQ		
	complex I intermediate associated protein 30	SSSSSSSTPTTT	Clavicipitaceae	EfM3.067320
	bZIP transcription factor	QQQPQQPQQQQPHHHQQQQQ	Clavicipitaceae	EfM3.072790
	ribosomal protein L24E	HQQHHHQAHQQHHQAHQQHQAHHHQQQQQQQQQQQQQ	Clavicipitaceae	EfM3.078900
		QQQQQQHH		
	putative sulfide quinine reductase	HHHHHQHQHHH	*Epichloë*	EfM3.051760
	hypothetical protein	QQQQQQQQQQQQQQQQQQQQQQQQQAAAQA	Sordariomycetes	EfM3.013050
(CAG)_5_(CAA)_5_	nucleoporin NIC96	QQQQQQQQQQQQQ	Sordariomycetes	EfM3.043780
		QQQQQQQQQQ		
	transcriptional corepressor cyc8	QQQQQQQQQQQQQQQQQQQQQQQQQQQ	Sordariomycetes	EfM3.057580
	75 gamma secalin	QQQQQQ	Sordariomycetes	EfM3.081630
		QQQQQQQQQQQQHQ		
		PEPEPEPEPEPEPEPEPEPE		
	transmembrane anterior posterior transformation I/ putative cytomegalovirus gH-receptor family protein	QQQQQQQQQQQQQ	Clavicipitaceae	EfM3.010720
	C6 zinc finger domain containing protein	QQQQQQQQQQQQQAAAA	Sordariomycetes	EfM3.043620
	topoisomerase II associated protein	QQQQQQQQQQHQQQ	Sordariomycetes	EfM3.017660
		QQQQQQQQQQQQQQQHQQQQQQHHQHQQQHHQQQQLQQQ		
		QQQQQGQQQQ		
		QQQQQQQHQQ		
		AAAAAAAA		

^1^Clavicipitacae includes *Epichloë*, *Claviceps*, *Periglandula*; Sordariomycetes includes *Epichloë*, *Claviceps*, *Periglandula*, *Fusarium*, *Magnaporthe*, *Neurospora*.

## Discussion

The use of SSR markers in diagnostic identification of fungal species is still widely employed for both rapid pathogen identification in response to incursions, and for analysis of population genetic diversity [34,35,36,37]. These markers are also widely used for identification of *Epichloë* endophytes in *Pooideae* grasses [38,39,40]. However, despite the substantial use of SSR markers in species identification, relatively little is known about their genomic location or impacts on gene structure. We show here that the extensively utilized *Epichloë* B10 SSR [38,39,40] is highly polymorphic, not only in sequence length but also tri-nucleotide structure. For example, *E*. *amarillans* E57 and *E*. *typhina* E8 shared a B10 allele of the same length, yet the repeat structure within was completely different. Specifically, *E*. *amarillans* had a shorter CAG repeat but a longer CAA repeat than *E*. *typhina*. These differences highlight that the species resolving power of this repeat lies not only in the polymorphic length but also in the repeat sequence.

The added resolving power of the B10 SSR sequence can be extended to identification of the parental origins of *Epichloë* interspecific hybrids. Hybrid parentage is usually determined by phylogenetic analysis [41,42]. Here we confirm using the sequences of B10 SSRs that the interspecific hybrid *E*. *uncinata* [11,13], has originated by hybridization of *E*. *typhina* and *E*. *bromicola*. This approach, therefore, provides promise as a novel method for determination of interspecific hybrid parentage. Sequencing of the B10 SSR also allowed us to distinguish between different *E*. *uncinata* ecotypes, previously only differentiated based on phenotypic characteristics.

Analysis of the genomic location of the B10 SSR revealed that it was located within the coding region of a putative bZIP transcription factor. Although SSRs are found within protein-coding regions, they are generally much more abundant in non-coding regions [43]. Tandem repeats within coding regions of fungal genes have been proposed to rapidly increase the rate of protein evolution and allow faster adaptation to environmental change [44]. Homologues of the putative bZIP transcription factor are found across the Sordariomycetes, and are highly conserved at the amino acid level. However, the presence of the B10 SSR exclusively within the Clavicipitaceae family lead to insertion of a glutamine rich repeat with a histidine core. Polyglutamine repeats within transcription factors, often caused by SSRs, have been shown to increase transcriptional activation, and are speculated to play a role in evolutionary modulation of transcription factor activity [45].

A search of the *Epichloë* genome for additional B10-like repeats within coding regions identified a further nineteen SSR-interrupted genes, spanning a variety of gene families. Many of these were restricted to the Clavicipitaceae family. A B10-like repeat, designated B12, was also identified within the coding region of a putative transcription factor found exclusively within *Epichloë* species. Similar to what was found for the putative bZIP transcription factor, this interruption resulted in a glutamine rich repeat with a histidine core.

In the opportunistic human pathogen *Candida albicans*, the CAI SSR lies within the coding region of the transcription factor *RLM1*, and variation in SSR length affects the response of *C*. *albicans* to various stresses [46]. Similarly, variations in the length of an SSR within the coding region of the *Saccharomyces cerevisiae* MAP kinase *SLT2*, leading to expansion of a glutamine rich repeat, has been proposed to allow *S*. *cerevisiae* to adapt rapidly to environmental change [47]. These observations that variation in SSR length can lead to phenotype modulation lead to the hypothesis that variation in SSRs within *Epichloë* species may play a role in the response of these endophytes to their immediate environment–their host plant. *Epichloë* endophytes are very host specific [48,49], which potentially could be due, at least in part, to variation in SSRs contained within coding regions.

In conclusion, our study has uncovered new potential for the use of SSRs in enhanced species identification, including ancestry reconstruction of hybrid species, by combining SSR length polymorphism with SSR sequence information. We have also identified a potentially important role for SSRs in the evolution of a number of *Epichloë* genes, which may play a role in the fungal response to the host, and are ideal candidates for further functional characterization.

## Supporting information

S1 Fig Amino acid sequence alignment of putative bZIP transcription factors. CLUSTALW alignment of amino acid sequences showing the putative bZIP domain (red box) and the B10 SSR region (green box). The SSR is seen as repeated glutamine (Q) and histidine (H) residues. Gene IDs with associated GenBank protein accession numbers or gene models of protein homologues are given in the legend of Fig 3.
(TIF)

S2 Fig RT-PCR analysis of the B10 SSR in *Epichloë typhina* E8. (a) Gel electrophoresis of a fragment of the bZIP transcription factor amplified from gDNA and cDNA. M, 1 kb+ ladder. (b) DNA chromatogram of sequenced cDNA showing the B10 SSR within the exonic region.
(TIF)

S3 Fig Amino acid sequence alignment of putative copper sensing transcription factors. CLUSTALW alignment of amino acid sequences showing the putative copper-fist domain (red box) and the B12 SSR region (green box). The SSR is seen as repeated glutamine (Q) and histidine (H) residues. Gene IDs with associated GenBank protein accession numbers or gene models of protein homologues are given in the legend of Fig 6.
(TIF)

S4 Fig Sequence analysis of PCR-amplified B12 SSR in *Epichloë uncinata* strains. Comparison of PCR-amplified B12 SSR sequences from *E*. *uncinata* U2, U3, U4, U5 and U6 strains with the B12 SSR sequences from *E*. *typhina* and *E*. *bromicola*.
(TIF)

S5 Fig Amino acid sequence alignment of putative G-protein coupled receptors. CLUSTALW alignment of amino acid sequences showing the B13 SSR region (green box). The SSR is seen as repeated glutamine (Q) and histidine (H) residues. Gene IDs with associated GenBank protein accession numbers or gene models of protein homologues are given in the legend of Fig 7.
(TIF)

S1 Table Fungal strains. (DOCX)

S2 Table Primers used in this study. (DOCX)
