# Supplementary figures and images for: Analysis of simple sequence repeat (SSR) structure and sequence within Epichloë endophyte genomes reveals impacts on gene structure and insights into ancestral hybridization events
Source: PLoS One. 2017 Sep 8;12(9):e0183748. doi: 10.1371/journal.pone.0183748 (PMC5590859; doi:10.1371/journal.pone.0183748)

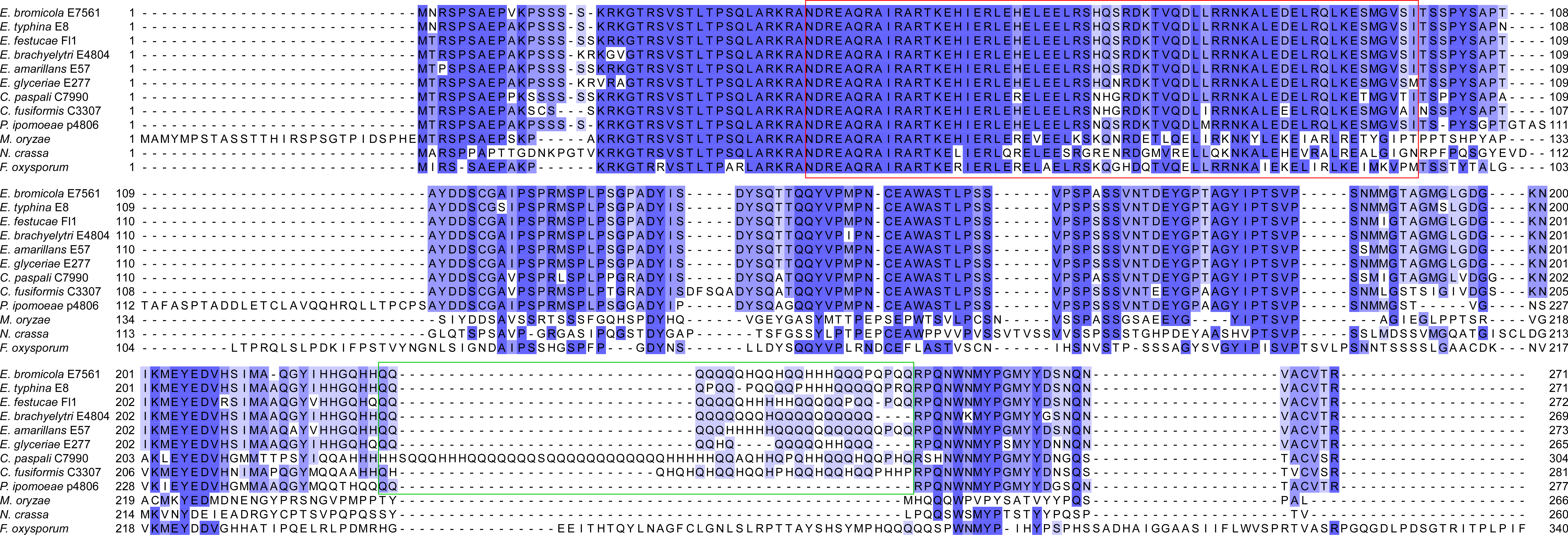

Supplement: S1 Fig — CLUSTALW alignment of amino acid sequences showing the putative bZIP domain (red box) and the B10 SSR region (green box). The SSR is seen as repeated glutamine (Q) and histidine (H) residues. Gene IDs with associated GenBank protein accession numbers or gene models of protein homologues are given in the legend of Fig 3. (TIF) [file pone.0183748.s001.tif]

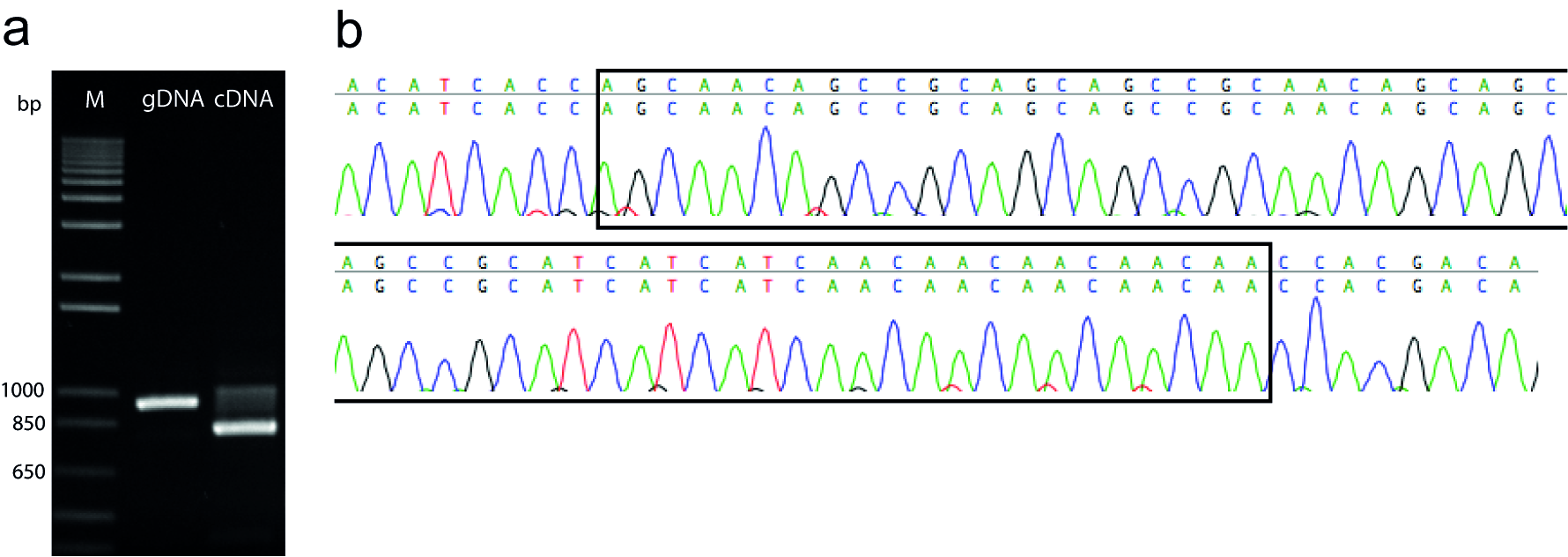

Supplement: S2 Fig — (a) Gel electrophoresis of a fragment of the bZIP transcription factor amplified from gDNA and cDNA. M, 1 kb+ ladder. (b) DNA chromatogram of sequenced cDNA showing the B10 SSR within the exonic region. (TIF) [file pone.0183748.s002.tif]

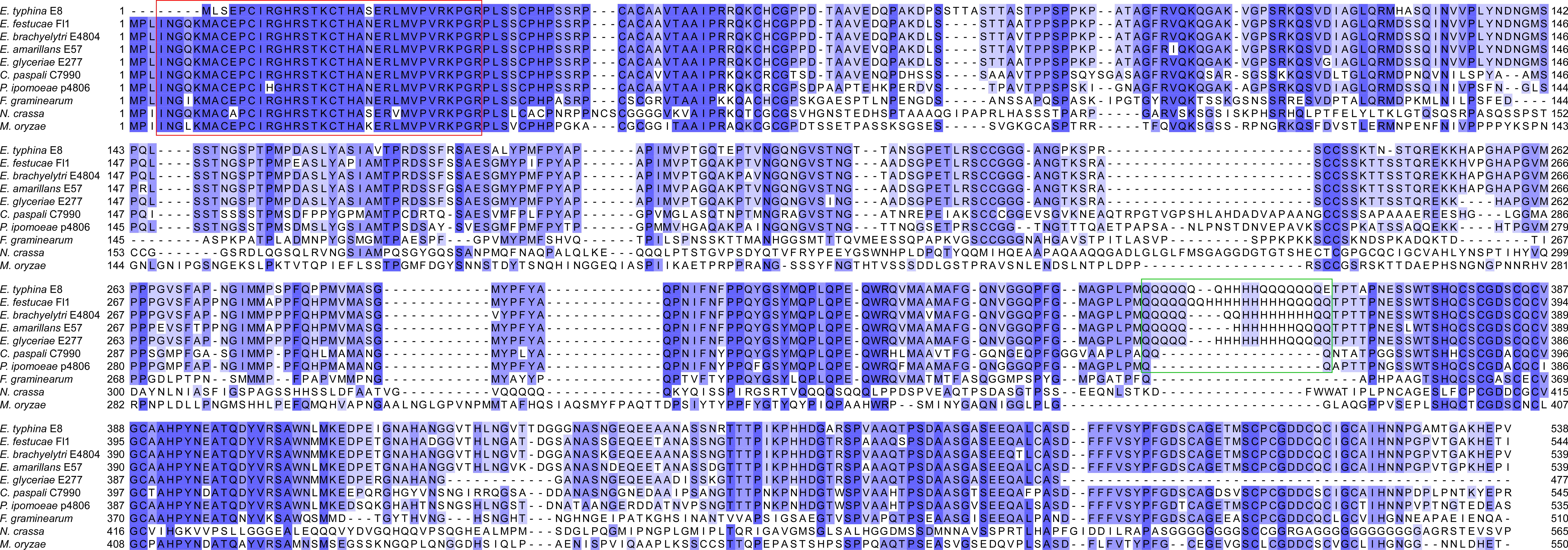

Supplement: S3 Fig — CLUSTALW alignment of amino acid sequences showing the putative copper-fist domain (red box) and the B12 SSR region (green box). The SSR is seen as repeated glutamine (Q) and histidine (H) residues. Gene IDs with associated GenBank protein accession numbers or gene models of protein homologues are given in the legend of Fig 6. (TIF) [file pone.0183748.s003.tif]

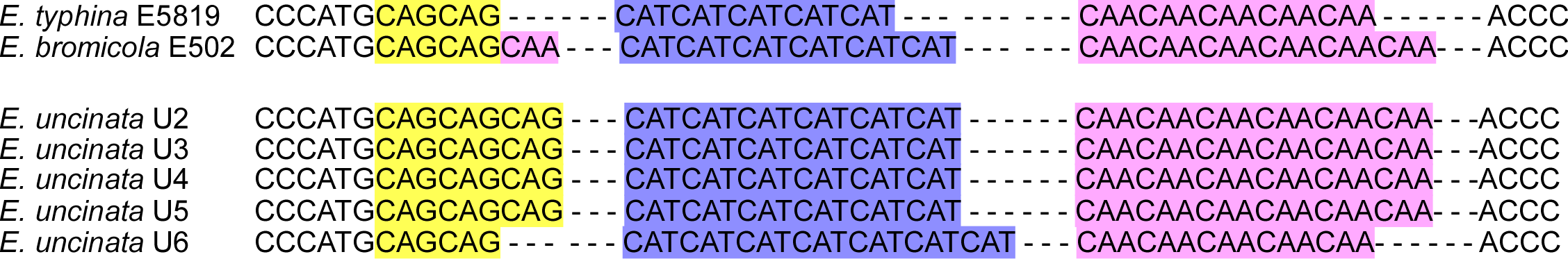

Supplement: S4 Fig — Comparison of PCR-amplified B12 SSR sequences from E. uncinata U2, U3, U4, U5 and U6 strains with the B12 SSR sequences from E. typhina and E. bromicola. (TIF) [file pone.0183748.s004.tif]

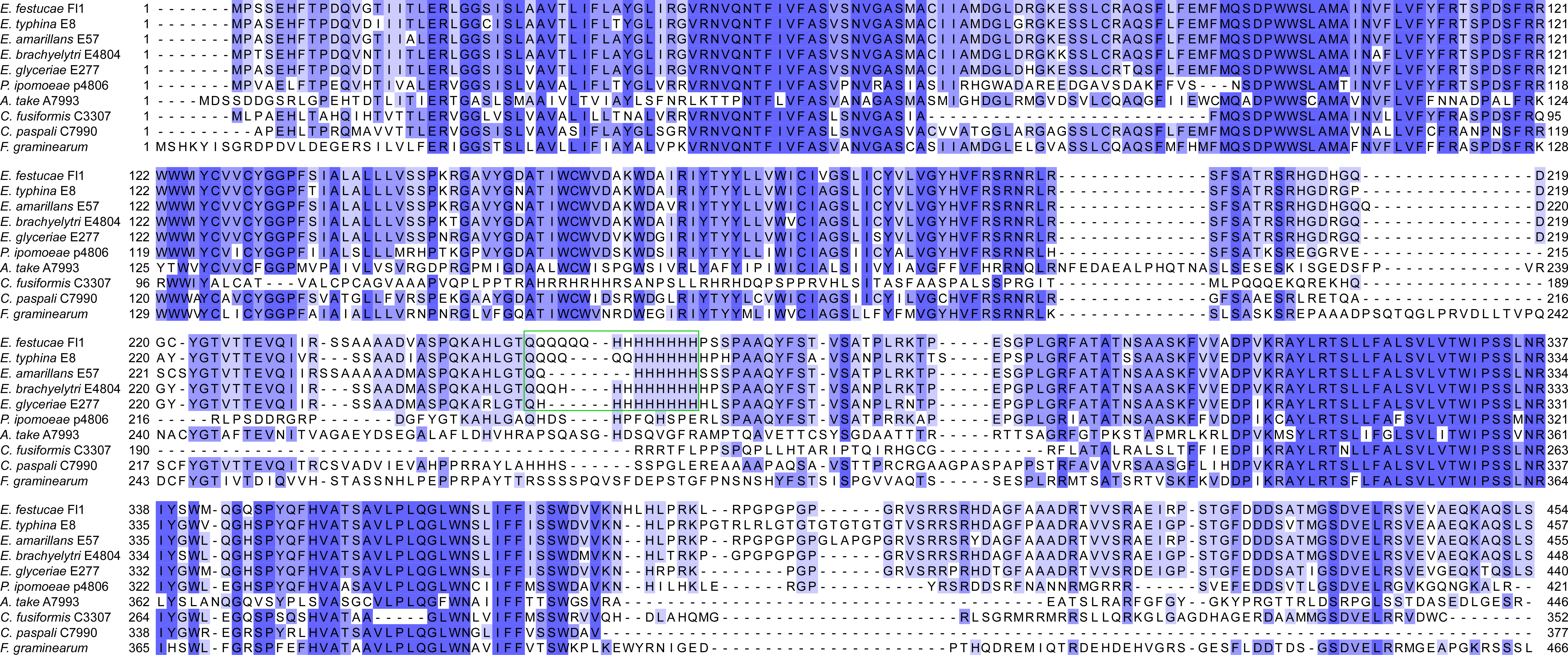

Supplement: S5 Fig — CLUSTALW alignment of amino acid sequences showing the B13 SSR region (green box). The SSR is seen as repeated glutamine (Q) and histidine (H) residues. Gene IDs with associated GenBank protein accession numbers or gene models of protein homologues are given in the legend of Fig 7. (TIF) [file pone.0183748.s005.tif]
