# Supplementary material for: Analysis of simple sequence repeat (SSR) structure and sequence within Epichloë endophyte genomes reveals impacts on gene structure and insights into ancestral hybridization events
Source: PLoS One. 2017 Sep 8;12(9):e0183748. doi: 10.1371/journal.pone.0183748 (PMC5590859; doi:10.1371/journal.pone.0183748)
Supplement: S1 Table — (DOCX) [file pone.0183748.s006.docx]

**S1 Table. Fungal strains.**

| **Fungal strain** | **Strain identifier** | **Relevant characteristics** | **References** |
| --- | --- | --- | --- |
| *Epichloë typhina* | E8 | Wild-type | [1] |
| *Epichloë bromicola* | E799 | Wild-type | [1] |
| *Epichloë uncinata* | PN2807 | Wild-type U2 | This study |
| *Epichloë uncinata* | PN2809 | Wild-type U12 | This study |
| *Epichloë uncinata* | PN2810 | Wild-type U3 | This study |
| *Epichloë uncinata* | PN2811 | Wild-type U4 | This study |
| *Epichloë uncinata* | PN2812 | Wild-type U6 | This study |
| *Epichloë uncinata* | PN2813 | Wild-type U10 | This study |
| *Epichloë uncinata* | PN2815 | Wild-type U13 | This study |
| *Epichloë uncinata* | PN2816 | Wild-type U5 | This study |
| *Epichloë uncinata* | PN2817 | Wild-type U7 | This study |
| *Epichloë uncinata* | PN2820 | Wild-type U9 | This study |

**References:**

1. Leuchtmann A, Schardl CL. Mating compatibility and phylogenetic relationships among two new species of *Epichloë* and other congeneric European species. Mycol Res. 1998; 102(10):1169-82.
