# Supplementary material for: Analysis of simple sequence repeat (SSR) structure and sequence within Epichloë endophyte genomes reveals impacts on gene structure and insights into ancestral hybridization events
Source: PLoS One. 2017 Sep 8;12(9):e0183748. doi: 10.1371/journal.pone.0183748 (PMC5590859; doi:10.1371/journal.pone.0183748)
Supplement: S2 Table — (DOCX) [file pone.0183748.s007.docx]

**S2 Table. Primers used in this study.**

| **Primer name** | **Sequence (5’-3’)** | **Purpose** |
| --- | --- | --- |
| B10.1 | CGCTCAGGGCTACATACACCATGG | Amplify B10 |
| B10.2 | CTCATCGAGTAACGCAGGCGACG | Amplify B10 |
| B11.1 | CATGGATGGACAAGAGATTGCACG | Amplify B11 |
| B11.4 | TTCACTGCTACAATTCTGTCCAGC | Amplify B11 |
| B12.1 | TGTAGGAGGCCAACCTTTTG | Amplify B12 |
| B12.2 | CTTGTTCACCACTCGAAGCA | Amplify B12 |
| bZ1.1 | ATGAATCGATCACCATCAGC | Expression analysis of B10 |
| bZ1.2 | CATCGAGTAACGCAGGCGAC | Expression analysis of B10 |
| bZ1.3 | AAACGAAAGGGCACCCGAAG | Expression analysis of B10 |
| M13F | TGTAAAACGACGGCCAGT | Sequencing |
| M13R | GAGCGGATAACAATTTCACACAG | Sequencing |
